# Pterostilbene on Metabolic Parameters: A Randomized, Double-Blind, and Placebo-Controlled Trial

**DOI:** 10.1155/2014/459165

**Published:** 2014-06-25

**Authors:** Daniel M. Riche, Krista D. Riche, Chad T. Blackshear, Corey L. McEwen, Justin J. Sherman, Marion R. Wofford, Michael E. Griswold

**Affiliations:** ^1^The University of Mississippi School of Pharmacy, 2500 North State Street, Jackson, MS 39216, USA; ^2^The University of Mississippi Medical Center, 2500 North State Street, Jackson, MS 39216, USA; ^3^Department of Pharmacy Practice, University of Mississippi School of Pharmacy, 2500 North State Street, Jackson, MS 39216, USA; ^4^Department of Medicine, University of Mississippi Medical Center, 2500 North State Street, Jackson, MS 39216, USA; ^5^St. Dominic-Jackson Memorial Hospital, 969 Lakeland Drive, Jackson, MS 39216, USA; ^6^Center of Biostatistics, University of Mississippi Medical Center, 2500 North State Street, Jackson, MS 39216, USA; ^7^Cleveland Clinic, 9500 Euclid Avenue, Cleveland, OH 44195, USA

## Abstract

*Introduction*. The purpose of this trial was to evaluate the effect of pterostilbene on metabolic parameters. *Methods*. A prospective, randomized, double-blind, and placebo-controlled study that enrolled 80 patients with a total cholesterol ≥200 mg/dL and/or LDL ≥ 100 mg/dL. Subjects were divided into four groups: (1) pterostilbene 125 mg twice daily; (2) pterostilbene 50 mg twice daily; (3) pterostilbene 50 mg + grape extract (GE) 100 mg twice daily; (4) matching placebo twice daily for 6–8 weeks. Endpoints included lipids, blood pressure, and weight. Linear mixed models were used to examine and compare changes in parameters over time. Models were adjusted for age, gender, and race. *Results*. LDL increased with pterostilbene monotherapy (17.1 mg/dL; *P* = 0.001) which was not seen with GE combination (*P* = 0.47). Presence of a baseline cholesterol medication appeared to attenuate LDL effects. Both systolic (−7.8 mmHg; *P* < 0.01) and diastolic blood pressure (−7.3 mmHg; *P* < 0.001) were reduced with high dose pterostilbene. Patients not on cholesterol medication (*n* = 51) exhibited minor weight loss with pterostilbene (−0.62 kg/m^2^; *P* = 0.012). *Conclusion*. Pterostilbene increases LDL and reduces blood pressure in adults. This trial is registered with Clinicaltrials.gov NCT01267227.

## 1. Introduction

Metabolic syndrome (MetS) refers to a cluster of risk factors including increased cholesterol concentrations, high blood pressure, larger waist circumference, and elevated blood glucose. Based on the National Health and Nutrition Examination Survey, 34% of adults 20 and older meet the criteria for MetS [1]. The individual components of MetS are risk factors for both cardiovascular disease (CVD) and type 2 diabetes mellitus (T2DM). It is estimated that 83.6 million American adults (more than 1 in 3) have at least one type of CVD [2]. Mortality data from 2009 has shown that nearly 1 in every 3 deaths in the United States lists CVD as an underlying condition [3]. Additionally, 1 out of every 6 hospital stays results from CVD with an estimated health-care cost of $71.2 billion, approximately 1/4 of the total cost of inpatient hospital care in the United States [2].

Diet-derived phenols represent an attractive treatment modality for many different disease states. Recently, the Nurses' Health Study reported a relationship between anthocyanin-rich foods (i.e., blueberries) and reduced risk of myocardial infarction (MI) in >90,000 women [4]. There also appeared to be a relationship between the quantity of anthocyanin intake and MI, indicating a potential dose-dependent reduction in heart disease [4].

Pterostilbene, a phenol chemically related to resveratrol, is a naturally occurring phytoalexin found in blueberries, grapes, and various other plants. Previous studies have demonstrated that pterostilbene possesses multiple pharmacologic properties, including hypolipidemic, antidiabetic, and anti-inflammatory mechanisms [5, 6]. Phenols, such as resveratrol and pterostilbene, are thought to contribute to the CVD protection provided by red wine [7].

Peroxisome proliferator activated receptor alpha isoforms (PPAR-*α*), found in the heart, liver, and muscles, exhibit pleiotropic effects including altering lipid metabolism [6, 8]. An* in vitro* analysis of resveratrol and its three analogues, including pterostilbene, evaluated PPAR-*α* activation. The investigators noted that pterostilbene demonstrates the highest induction of PPAR-*α*, with an 8- to 14-fold increase in activity relative to a control (ciprofibrate) [6]. This suggests that pterostilbene may be an effective PPAR-*α* agonist and thus a potent hypolipidemic agent. Additionally, pterostilbene may impact blood pressure. Pterostilbene has demonstrated attenuation of angiotensin converting enzyme, activation of several antioxidant pathways, and upregulation of nitric oxide synthase in the vascular endothelium, all of which are potential mechanisms for blood pressure reduction [9, 10].

Pterostilbene is structurally different from resveratrol as it only possesses 1 hydroxyl group. The remaining 2 hydroxyl groups in resveratrol are replaced with methoxy groups, increasing lipophilicity of pterostilbene [11]. This modification increases the oral bioavailability and lengthens the half-life of pterostilbene [11, 12].

The National Center for Complementary and Alternative Medicine (NCCAM) recognizes grape extract (GE) as a antioxidant. Procyanidin extract (via grape seed) has demonstrated normalization of blood pressure in pre- and mildly hypertensive patients via improvements in microcirculation [13]. In addition, a meta-analysis reported a decreased SBP over an average of <8 weeks amongst randomized trials evaluating grape seed extract [14]. While grape seed extract appears generally safe, the NCCAM lists “high blood pressure” as a potential side effect. GE was of particular interest in this study due to the potential for synergistic effects on blood pressure and oxidative stress.

Our trial is the first trial performed in humans evaluating the dose-ranging efficacy of pterostilbene with or without grape extract on metabolic parameters.

## 2. Materials and Methods

This trial was a prospective, randomized, double-blind, and placebo-controlled intervention trial. The target population was patients with hypercholesterolemia, defined as a baseline total cholesterol ≥200 mg/dL and/or baseline low-density lipoprotein cholesterol ≥100 mg/dL. Inclusion and exclusion criteria have been previously defined [15]. Both participants and care providers were blinded.

Eighty subjects were randomized in a 2 × 2 block design for presence of cholesterol medication into one of four groups: pterostilbene 50 mg twice daily (low dose), pterostilbene 125 mg twice daily (high dose), pterostilbene 50 mg/grape extract 100 mg twice daily (low dose + grape extract), or matching placebo by mouth twice daily for 6–8 weeks. A range was selected for the treatment period to allow participants flexible scheduling of final visits. Dose selection has been previously defined [15]. All patients received identical information on healthy lifestyle practices and counseling on compliance with currently prescribed medication regimens.

All clinical trial materials (including placebo) were supplied by Chromadex, Inc. Pterostilbene was provided in the form of pTeroPure, a >99% all-trans-pterostilbene. The specifications have been previously defined [16]. The grape extract (GE) was provided in the form of ShanStar Concord Grape, a bioflavonoid compound that contains no pterostilbene with a total phenolic content of 195–255 gallic acid equivalents mg/g by Folin-Ciocalteu method. The manufacturer was deemed in compliance with the Food & Drug Administration current good manufacturing practices prior to the initiation of this trial.

Efficacy parameters were collected at two visits (baseline and final). Primary efficacy measures included fasting lipid concentrations. Secondary measures included blood pressure and body weight. Measures of urinary oxidation are not described in this paper. All efficacy measures were stratified by presence of cholesterol medication at baseline. Donated blood was collected via venipuncture and analyzed at the University of Mississippi Pavilion Laboratory. Seated blood pressure was measured manually using mercury sphygmomanometer based on published measuring techniques for guidance [17]. Body weight was measured using a calibrated medical scale. Pill counts were utilized to assess for compliance.

Due to the release of the 2013 ACC/AHA Guidelines on the Treatment of Blood Cholesterol to Reduce Atherosclerotic Cardiovascular Risk in Adults, patient's atherosclerotic cardiovascular disease (ASCVD) risk scores and statin use appropriateness were determined and compared from baseline to final in each arm.

### 2.1. Ethics

This study was approved by the University of Mississippi Medical Center Institutional Review Board. The clinicaltrials.gov identifier is NCT01267227. All procedures were in accordance with the ethical standards set forth by the Helsinki Declaration of 1975 as revised in 1983.

### 2.2. Statistics

Linear mixed models were used for primary intention to treat (ITT) efficacy effects in order to account for intrasubject associations arising from the repeated measures before and after longitudinal design. The underlying missing-at-random architecture implicit in mixed models was assumed. Various models were fit to examine potential subgroup effects including as appropriate the following:

1. 3-way interaction models of final outcome × treatment group × baseline cholesterol medication status;
2. 3-way interaction models of final outcome × treatment group × baseline LDL status;
3. models assuming baseline value affected change similarly across treatment groups;
4. models assuming change in outcome were independent of baseline value (BMI).

Each model was examined in unadjusted and adjusted form (adjusting for age, race, and gender). The final reported treatment effects were obtained from the simplest appropriate adjusted model for each outcome. With sample sizes of 20 per treatment group and an assumed standard deviation of 18 mg/dL for LDL, we can statistically detect differences of 15.95 mg/dL between the pterostilbene treatments and placebo at the 5% significance level with 80% power. For new cholesterol guideline measures, a *t*-test was performed for continuous data (ASCVD risk score).

## 3. Results

**Figure 1:**
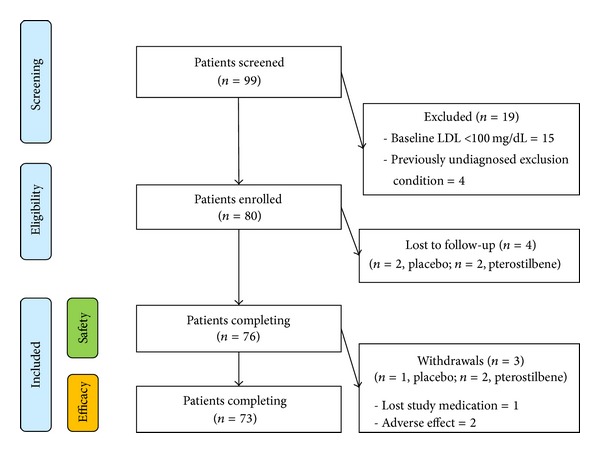
Enrollment strategy.

From January to December 2011, 80 patients (*n* = 20 per group) were enrolled (see Figure 1). Patient demographics are detailed in Table 1. The majority of patients completed the trial (91%) and demonstrated at least 80% compliance (81% of completers). There was an 8.8% overall attrition rate. The average study duration was 52 days.

**Figure 2:**
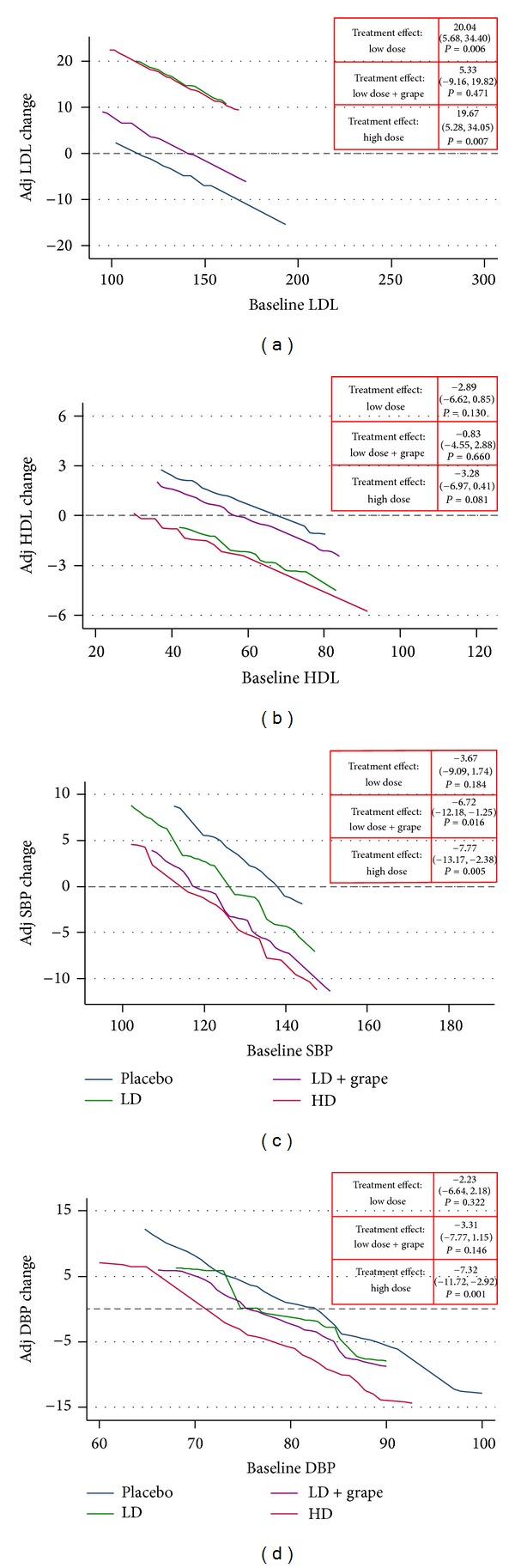
Efficacy analysis: lipids and blood pressure.* Interpretation.* Expected changes in an outcome (vertical axis) for any given level of baseline value (horizontal axis) across all four treatment groups. Adjusted for age, gender, and race. SBP: systolic blood pressure; DBP: diastolic blood pressure; LD: low dose; LD + Grape: low dose + grape combination; HD: high dose. Units: mg/dL or mmHg.

**Figure 3:**
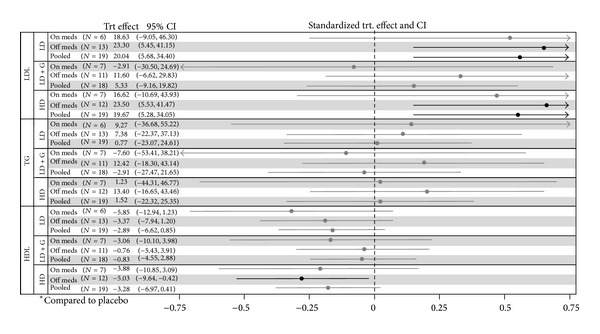
Lipid treatment effects by baseline cholesterol medication.* Interpretation.* Bold lines indicate significance. Significant measures to the right of 0 indicate an increased effect with the reported group versus placebo. Significant measures to the left of 0 indicate a decreased effect with the reported group versus placebo. Adjusted for age, gender, and race. TRT: treatment; CI: confidence interval; LD: low dose; LD + G: low dose + grape combination; HD: high dose. Units: mg/dL.

LDL increased with pterostilbene monotherapy (high dose and low dose groups combined) 17.1 mg/dL (*P* = 0.001), regardless of dose (see Figure 2). This increase was not significant in the GE combination group (*P* = 0.47). These findings were consistent regardless of baseline LDL ≤130 mg/dL versus >130 mg/dL. The presence of a baseline cholesterol medication appeared to attenuate this LDL increase in all groups (see Figure 3). As a function of the LDL increase, total cholesterol (TC) increased accordingly with both low dose and high dose pterostilbene (see Table 2). There was no significant change in HDL in the primary efficacy analysis. Subgroup analysis demonstrated a reduction of HDL with high dose pterostilbene monotherapy in patients not on cholesterol medication at baseline (−5.03 mg/dL; *P* = 0.033). There was no significant change in triglycerides across all groups.

There was a significant reduction versus placebo in SBP and DBP with high dose pterostilbene (see Figure 2). A reduction in SBP was also seen in the GE combination group (−6.72 mmHg; *P* = 0.016). The change in blood pressure appeared to be dose-dependent. There were no self-reported episodes of orthostatic hypotension or dizziness.

The average ASCVD risk scores are reported in Table 1. All treatment arms had similar ASCVD risk scores at baseline (*P* > 0.1 for all). Compared to baseline, there was no significant change in ASCVD risk score for any treatment arm (placebo: +0.59%, *P* = 0.33; high dose: +0.13%, *P* = 0.72; low dose: +0.02%, *P* = 0.96; GE combination: −0.83%, *P* = 0.11). Based on the 2013 ACC/AHA Guidelines on the Treatment of Blood Cholesterol to Reduce Atherosclerotic Cardiovascular Risk in Adults, appropriate statin use at baseline and final was 59% across all treatment arms. Only 2 patients demonstrated a change in ASCVD risk score that affected appropriate statin use. One patient receiving placebo had an ASCVD risk score increased >7.5% indicating the need for a statin; one patient receiving high dose pterostilbene had an ASCVD risk score decrease <7.5% indicating no need for a statin. Both of these patients were already on a statin.

**Figure 4:**
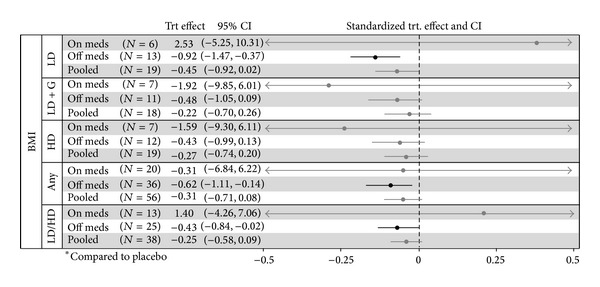
BMI treatment effects by baseline cholesterol medication.* Interpretation.* Bold lines indicate significance. Significant measures to the right of 0 indicate an increased BMI with the reported group versus placebo. Significant measures to the left of 0 indicate a decreased BMI with the reported group versus placebo. Adjusted for age, gender, and race. TRT: treatment; CI: confidence interval; LD: low dose; LD + G: low dose + grape combination; HD: high dose. Units: kg/m^2^.

There was no significant change in BMI in the primary efficacy analysis. Subgroup analysis demonstrated a decrease in BMI with (1) low dose, (2) pterostilbene monotherapy, and (3) overall population (all 3 groups combined) in patients not on cholesterol medication at baseline (see Figure 4).

Unadjusted models yielded the same efficacy results. Safety analysis, including blood glucose, has been previously reported [15]. Product purity was confirmed in a blinded, randomized assay upon completion of the trial [15].

## 4. Discussion

This is the first comparison of pterostilbene on metabolic parameters in humans. There appears to be a direct benefit of pterostilbene on both SBP and DBP. The reduction in SBP is comparable to other complementary and alternative medicine (CAM) regimens (including garlic, fish oil, and vitamin D). Although direct comparison studies have not been done, the reduction in DBP seems to surpass most CAM therapies (including coenzyme Q10, vitamin C, and melatonin) [18, 19].

The change in lipid parameters is contradictory to those demonstrated in animal models. LDL increased in both the low dose and the high dose pterostilbene groups. The effect was not seen in the GE combination arm. There was also no increased LDL seen with the presence of baseline cholesterol medication. The proposed mechanism of action of pterostilbene is PPAR-*α* agonism, a transcription factor that regulates lipid metabolism in various ways [5]. FDA-approved PPAR agonists (e.g., pioglitazone, rosiglitazone, and fenofibrate) have reported increases in LDL cholesterol in randomized, controlled trials. Traditional PPAR-*γ* agonists, thiazolidinediones, have consistently demonstrated LDL increases. The GLAI study reported similar LDL increases (12.3–21.3 mg/dL with pioglitazone and rosiglitazone, resp.) as seen with pterostilbene monotherapy [20]. Fenofibrate is a more selective PPAR-*α* agonist that has demonstrated a variability in regard to LDL. It is also known that fenofibrate has the potential to increase LDL, particularly in the setting of severe hypertriglyceridemia [21]. The causal factor of pterostilbene on increasing LDL remains unclear, but cross-selectivity with PPAR-*γ*, increased catabolism of triglyceride-rich lipoproteins, and/or gene-transcription related factors could be investigated.

As a PPAR-*α* agonist, pterostilbene would be expected to have the most profound effect on TG as a lipid marker; however, there was no significant result related to TG in any group. Fenofibrate is consistently associated with substantial decreases in serum TG (20–50%), which is usually directly proportional to baseline TG [21]. Considering the average baseline TG concentrations in this study were <165 mg/dL (and as low as 110 mg/dL in the low dose group), no change is an expected outcome. A study assessing the effect of pterostilbene on elevated TG (baseline 200–499 mg/dL) may be warranted. Pterostilbene does not appear to significantly affect HDL. Interestingly, the reduction in BP with high dose pterostilbene is similar to that seen with selective PPAR-*γ* agonists [22].

With the advent of the 2013 ACC/AHA Guidelines on the Treatment of Blood Cholesterol to Reduce Atherosclerotic Cardiovascular Risk in Adults, recommendations related to cholesterol management have changed significantly [23]. The expert panel reports that there is no randomized, controlled trial evidence to support specific LDL treatment targets [23]. Rather, the impetus of treatment is based on appropriate statin use. Logically, marginal changes to LDL are only relevant if it leads to an increase in a patient's ASCVD risk score from baseline eliciting the need for a statin. Results demonstrate that despite LDL increases, there is no significant change from baseline in ASCVD risk score or appropriate statin use regardless of treatment. The primary reason for the lack of change in overall ASCVD risk score is likely due to the decrease demonstrated in systolic blood pressure. In contrast, the 2014 Evidence-Based Guidelines for the Management of High Blood Pressure in Adults detail goal-oriented recommendations for the treatment and management of hypertension (HTN) [24]. Therefore, reductions in blood pressure demonstrated with high dose pterostilbene could play a role in the management of HTN.

The dose-dependent nature of pterostilbene's effect on blood pressure mirrors the hypothesis of reduced MI incidence with anthocyanin. As BP decreases due to high dose pterostilbene, the change in SBP and DBP from baseline reaches zero at 114 ± 12 mmHg and 70 ± 5 mmHg, respectively. Thus, pterostilbene would not be expected to cause hypotension or symptomatic orthostasis in normotensive patients. Accordingly, orthostatic hypotension was not reported as an adverse effect in this study. Upon visual inspection of Figure 2, it is interesting to note that patients in the prehypertension range (SBP = 120–139 mmHg or DBP = 80–89 mmHg) appeared to have increasing blood pressure over time in the placebo group. Due to the short duration of this trial, investigation for pterostilbene as an option to delay conversion from prehypertension to HTN is warranted. GE combination demonstrated a reduction in SBP with a confidence interval consistent with meta-analysis results (−1.54 mmHg in SBP) evaluating GE for HTN [14]. This finding gives confidence to the blood pressure measurement technique used in the study.

Consistent with the selective nature of PPAR-*α* agonists, pterostilbene is overall weight neutral. There was significant weight loss in certain subgroups. As previously reported in the safety analysis, participants indicating an increased appetite (*n* = 4) gained an average 1.7 pounds [15]. This finding coupled with similar LDL increases and BP reduction may indicate cross-selectivity for PPAR-*γ* activation with pterostilbene in certain patients. This study was not powered to determine weight changes in a single arm; therefore, a larger study isolating weight-related endpoints in a controlled manner should be conducted.

Systemic exposure (e.g., plasma concentration) was not measured in this study. At the time of this study, there was little known about plasma concentrations of pterostilbene in humans (e.g., reference ranges). Plasma concentrations can infer that absorption occurred but not prove pharmacological bioavailability at the site of action. In the absence of plasma concentrations, placebo-compared changes are appropriate for assessment of potential cause/effect relationships. There was a high rate of patient compliance with the study regimens. The dose-dependent nature of blood pressure effect indicates that adequate product exposure occurred in the treatment groups. The linearity of a cause/effect relationship with plasma exposure should be evaluated in humans.

Some limitations in this study include a small sample size, single center, and short trial duration. While lack of automated and 24-hour ambulatory blood pressure monitoring can be considered a limitation, manually measured in-office blood pressure is currently the standard of care for clinical trials [25]. There were 3 patients (1 placebo, 2 high doses) who stopped their statin medication during the course of the study against medical advice. Although LDL increased in all 3 cases, exclusion of these data did not impact the significance of reported LDL measures.

## 5. Conclusion

Pterostilbene increases LDL when used in monotherapy. Pterostilbene reduces blood pressure in adults at 250 mg/day doses. There appears to be potential for weight reduction in certain subgroups with pterostilbene. Future studies should evaluate high dose pterostilbene with GE in a hypertensive population.

## Figures and Tables

**Table 1:** Baseline demographics.

Characteristic	Placebo∗ (*n* = 20)	Low dose (*n* = 20)	Low dose + GE (*n* = 20)	High dose (*n* = 20)
Age (years)	54.40 (11.88)	53.55 (7.90)	52.95 (13.73)	53.55 (11.17)
Female	13 (65%)	15 (75%)	15 (75%)	14 (70%)
Race				
Caucasian	10 (50%)	15 (75%)	15 (75%)	16 (80%)
African American	10 (50%)	5 (25%)	3 (15%)	4 (20%)
Asian	0 (0%)	0 (0%)	2 (10%)	0 (0%)
Weight (lbs)	194.66 (46.10)	185.32 (50.91)	192.76 (44.56)	181.79 (40.56)
BMI (kg/m^2^)	30.09 (6.30)	30.30 (8.29)	31.09 (5.86)	29.25 (6.02)
Blood pressure				
SBP (mmHg)	130.20 (15.21)	125.15 (14.35)	125.40 (14.01)	128.75 (19.99)
DBP (mmHg)	80.95 (8.89)	78.70 (5.59)	78.50 (7.32)	78.95 (10.16)
Hypertensive	13 (65%)	12 (60%)	8 (40%)	11 (55%)
Cholesterol				
LDL (mg/dL)	143.80 (44.03)	142.35 (29.46)	140.90 (37.03)	140.20 (27.41)
HDL (mg/dL)	53.25 (16.01)	63.30 (18.17)	56.60 (15.10)	58.25 (23.41)
Triglycerides (mg/dL)	118.45 (55.02)	109.95 (45.40)	163.90 (70.57)	124.50 (82.94)
Cholesterol medication	8 (40%)	7 (35%)	7 (35%)	7 (35%)
Statin	6 (30%)	6 (30%)	7 (35%)	6 (30%)
Smokers	0 (0%)	3 (15%)	2 (10%)	3 (15%)
ASCVD risk score (%)	6.8 (5.1)	7.5 (11.6)	7.5 (10.1)	8.6 (7.6)
Framingham 10-year risk (%)	5.70 (6.87)	5.50 (7.27)	6.40 (8.80)	5.80 (5.88)

*Values are mean (SD) or *n* (%).

BMI: body mass index; LDL: low-density lipoprotein; HDL: high-density lipoprotein; TG: triglycerides; ASCVD: atherosclerotic cardiovascular disease.

*denote that these data are “Compared to Placebo”.

**Table 2:** Body weight and additional lipid efficacy results.

Outcome	LD		LD + GE		HD	
	Effect (95% CI)	*P* value	Effect (95% CI)	*P* value	Effect (95% CI)	*P* value
BMI	−0.27 (−0.74, 0.20)	*P* = 0.268	−0.19 (−0.64, 0.26)	*P* = 0.407	−0.26 (−0.70, 0.18)	*P* = 0.250
TC	**18.10 (2.19**, **34.00)**	*P* = 0.026	4.56 (−11.50, 20.63)	*P* = 0.578	**16.39 (0.49**, **32.30)**	*P* = **0.043**
TG	0.77 (−23.07, 24.61)	*P* = 0.949	−2.91 (−27.47, 21.65)	*P* = 0.816	1.52 (−22.32, 25.35)	*P* = 0.901

*Compared to placebo.

**Bold indicates significance.

BMI: body mass index (kg/m^2^); TC: total cholesterol (mg/dL); LDL: low-density lipoprotein (mg/dL); TG: triglycerides (mg/dL); HDL: high-density lipoprotein (mg/dL); LD: low dose; LD + Grape: low dose + grape combination; HD: high dose.
